# The alcohol flushing response is associated with the risk of depression

**DOI:** 10.1038/s41598-022-16276-2

**Published:** 2022-07-22

**Authors:** Seonghee Jeon, Heewon Kang, Inhyung Cho, Sung-il Cho

**Affiliations:** 1 Department of Public Health Science, Graduate School of Public Health, Seoul National University, Seoul, Korea; 2 Institute of Health and Environment, Graduate School of Public Health, Seoul National University, 1 Gwanak-ro, Gwanak-gu, Seoul, 08826 Republic of Korea

**Keywords:** Depression, Biomarkers, Risk factors

## Abstract

The alcohol flushing response is experienced by 36–45% of East Asians after they consume a small amount of alcohol. Because individuals with this response are unable to metabolize the toxic acetaldehyde derived from alcohol effectively, the response offers a potential indicator of the health risks associated with alcohol intake. Depression is a major health problem linked to alcohol consumption; it might also be associated with the alcohol flushing response. Therefore, we examined the association between the alcohol flushing response and the risk of depression in the general population of South Korea. Our analysis included 139,380 participants and used data from the 2019 Korean Community Health Survey. Only current drinkers were considered in the analysis. The relationship between the alcohol flushing response and depression was evaluated by logistic regression analysis using SAS 9.4. Of the participants, more than one-third were current flushers; compared to never flushers, current flushers had a significantly greater risk of depression (adjusted odds ratio [AOR] 1.23, 95% confidence interval [CI] 1.12–1.34, *P* < 0.001). Former flushers did not exhibit a risk of depression. The risk of depression was significantly greater among alcohol flushers who drank < 15 g alcohol/day (< 5 g alcohol/day: AOR 1.20, 95% CI 1.07–1.35, *P* = 0.002; 5–14.9 g alcohol/day: AOR 1.39, 95% CI 1.13–1.70, *P* = 0.002). In conclusion, a large number of South Koreans experience the alcohol flushing response; compared with never flushers, current flushers are more likely to develop depression with a small dose of alcohol (< 15 g alcohol/day).

## Introduction

The alcohol flushing response (i.e., “Asian flush”) is observed in 36–45% of East Asians (Koreans, Chinese, and Japanese)^1,2^; it occurs in carriers of mutant ALDH2*2 alleles that render ALDH2 inactive. Because ALDH2 encodes the enzyme that eliminates the toxic acetaldehyde derived from alcohol, ALDH2*2 individuals demonstrate slow or no metabolism of acetaldehyde^3^. Inactive ALDH2 homozygotes (ALDH2*2/*2) lack detectable ALDH2 activity, whereas low-activity ALDH2 heterozygotes (ALDH2*1/*2) exhibit a 100-fold reduction of ALDH2 activity^4^. Consequently, ALDH2*2 is linked to a significant increase in the blood acetaldehyde level after alcohol consumption^5^. After the consumption of alcohol (even a small amount), acetaldehyde accumulates in excess; this induces a physiological response in the face, which is known as the alcohol flushing response^1^. Individuals with the alcohol flushing response are susceptible to the risks of alcohol intake because they are less capable of metabolizing acetaldehyde, compared to individuals without the response (i.e., non-flushers or never flushers)^6–9^.

Since the alcohol flushing response is an indicator of internal acetaldehyde exposure associated with alcohol consumption, this response may be related to the risk of depression. Acetaldehyde is associated with depressive states, either through direct or indirect pathways. Stress-related peptide interactions induced by acetaldehyde intoxication may trigger a depressive state^10^. Excessive acetaldehyde accumulation can also lead to alcohol-use disorder (AUD)-associated depression. Acetaldehyde triggers dopamine release, and addictive behavioral traits are linked to increased dopamine levels in limbic regions^10^. The addictive behavioral traits are strongly associated with AUDs; this relationship can explain psychologically unstable states, such as depression^10,11^. Because individuals with the alcohol flushing response (i.e., flushers) are exposed to excessive acetaldehyde accumulation^1,2^, they may have an increased risk of acetaldehyde-associated depression despite low alcohol intake.

Although the association between the flushing response and depression has considerable importance, it has been addressed by few studies. Yoshimasu et al.^12^ proposed that the combination of ALDH2*1/*2 genotype and ADH1B*1/*1 genotype carries a significantly increased risk of depression. However, other studies have reported that protection from AUDs among individuals with inactive ALDH2 who have the flushing response; such individuals abstain from drinking or drink less than non-flushers^4,13^. This finding implies that alcohol flushers are less likely to experience AUD-associated depression^10^.

The association between alcohol flushing and depression may be influenced by alcohol intake, because flushers tend to drink less than non-flushers, thereby mitigating the effects of inactive ALDH2^14^. Flushers who drink heavily are generally considered rare and may be underrepresented. Most previous studies concerning the association of alcohol flushing and inactive ALDH2 with depression included small numbers of participants and did not adjust for alcohol intake.

The 2019 Korean Community Health Survey (KCHS) included questions about the alcohol flushing response. The survey was administered to more than 220,000 participants who were representative of the South Korean population; it provides an opportunity to investigate the association between the flushing response and depression, while adjusting for alcohol intake. Therefore, we used 2019 KCHS data to assess the prevalence of the alcohol flushing response among the South Korean population and determine its association with depression.

## Results

### Participant characteristics according to alcohol flushing status

Among the participants, 61.1% were never flushers, 34.8% were current flushers, and 4.1% were former flushers. Irrespective of flushing status, most of the participants were men, aged 40–59 years, with a fourth quartile family income level and non-obese, never smoker and no exerciser status, and who drank 1–2 drinks per occasion, drank < 5 g alcohol/day, had a drinking onset ≥ 19 years, and had never attempted to reduce or quit drinking alcohol. Most current flushers were with secondary education and drank monthly or less often, whereas most never and former flushers were with tertiary education and drank 2–4 times per month (Table 1).

**Table 1:** Participant characteristics according to alcohol flushing status (n = 139,380).

n (%)	Total	Never flusher	Former flusher	Current flusher	*P*-value
**Alcohol flushing status**					
Total	139,380 (100.0)	84,303 (61.1)	6112 (4.1)	48,965 (34.8)	< 0.001
**Sex**					
Male	73,847 (55.9)	44,230 (55.7)	3483 (58.6)	26,134 (55.9)	0.003
Female	65,533 (44.1)	40,073 (44.3)	2629 (41.4)	22,831 (44.1)	
**Age (years)**					
19–39	39,543 (39.3)	25,359 (41.2)	1137 (28.1)	13,047 (37.4)	< 0.001
40–59	57,477 (42.6)	34,945 (42.3)	2658 (47.9)	19,874 (42.6)	
60–69	24,383 (11.2)	13,775 (10.3)	1351 (15.2)	9257 (12.3)	
≥ 70	17,977 (6.8)	10,224 (6.2)	966 (8.7)	6787 (7.7)	
**Family income level**					
1st quartile	31,056 (14.4)	17,911 (13.7)	1624 (17.1)	11,521 (15.4)	< 0.001
2nd quartile	20,702 (12.8)	12,258 (12.5)	967 (13.7)	7477 (13.2)	
3rd quartile	39,548 (29.9)	23,984 (29.7)	1635 (29.5)	13,929 (30.2)	
4th quartile	48,074 (42.9)	30,150 (44.0)	1886 (39.7)	16,038 (41.2)	
**Educational level**					
None	6298 (1.8)	3783 (1.7)	347 (2.4)	2168 (1.8)	< 0.001
Primary	14,558 (5.3)	8142 (4.9)	842 (7.8)	5574 (5.8)	
Secondary	66,195 (45.9)	39,362 (45.0)	3111 (51.3)	23,722 (47.0)	
Tertiary	52,329 (47.0)	33,016 (48.5)	1812 (38.6)	17,501 (45.4)	
**Obesity**					
No (BMI < 25.0)	88,799 (64.6)	54,000 (64.8)	3796 (63.1)	31,003 (64.6)	0.118
Yes (BMI ≥ 25.0)	50,581 (35.4)	30,303 (35.2)	2316 (36.9)	17,962 (35.4)	
**Smoking status**					
Never smoker	80,657 (57.8)	49,471 (58.5)	3171 (52.3)	28,015 (57.1)	< 0.001
Ex-smoker	29,287 (19.8)	16,513 (18.7)	1549 (23.3)	11,225 (21.4)	
Current smoker	29,436 (22.4)	18,319 (22.8)	1392 (24.4)	9725 (21.5)	
**Exercise**					
No	103,359 (74.3)	62,673 (74.3)	4470 (74.0)	36,216 (74.3)	0.886
Yes	36,021 (25.7)	21,630 (25.7)	1642 (26.0)	12,749 (25.7)	
**Drinking frequency**					
Monthly or less	52,321 (36.5)	27,448 (31.0)	1781 (27.7)	23,092 (47.2)	< 0.001
2–4 times per month	42,100 (32.7)	26,430 (34.1)	1768 (32.6)	13,902 (30.3)	
2–3 times per week	29,616 (22.0)	20,143 (25.0)	1498 (25.4)	7975 (16.3)	
≥ 4 times per week	15,343 (8.8)	10,282 (9.9)	1065 (14.2)	3996 (6.3)	
**Drinks per occasion**					
1–2	50,320 (32.5)	26,381 (27.2)	1902 (26.9)	22,037 (42.5)	< 0.001
3–4	29,816 (21.1)	17,408 (20.1)	1373 (22.4)	11,035 (22.7)	
5–6	17,468 (13.3)	11,242 (14.0)	783 (13.9)	5443 (12.0)	
7–9	23,232 (17.8)	15,880 (20.2)	1159 (20.1)	6193 (13.1)	
≥ 10	18,544 (15.3)	13,392 (18.5)	895 (16.8)	4257 (9.6)	
**Alcohol intake (g/day)**					
< 5	84,890 (60.1)	46,878 (54.1)	3161 (51.0)	34,851 (71.5)	< 0.001
5–14.9	26,858 (20.5)	18,068 (23.0)	1335 (23.4)	7455 (15.8)	
15–29.9	17,520 (13.1)	12,344 (15.5)	946 (15.7)	4230 (8.6)	
≥ 30	10,112 (6.3)	7013 (7.3)	670 (9.9)	2429 (4.1)	
**Drinking onset**					
< 19 years old	36,499 (28.3)	22,459 (28.6)	1744 (30.2)	12,296 (27.6)	< 0.001
≥ 19 years old	102,881 (71.7)	61,844 (71.4)	4368 (69.8)	36,669 (72.4)	
**Attempts to reduce/quit drinking**					
No	119,543 (85.8)	71,956 (85.0)	4842 (79.1)	42,745 (87.9)	< 0.001
Yes	19,837 (14.2)	12,347 (15.0)	1270 (20.9)	6220 (12.1)	

Descriptive data are unweighted frequencies (n) with weighted percentages (%). 1 drink = 7 g alcohol.

BMI, body mass index (kg/m^2^).

### Association of the alcohol flushing response with depression

The prevalence of depression was highest among current flushers (weighted prevalence 2.93%, 2.66%, and 2.52% for current, former, and never flushers, respectively). The risk of depression was significantly greater among current flushers than among never flushers (adjusted odds ratio [AOR] 1.23, 95% confidence interval [CI] 1.12–1.34, *P* < 0.001). The analysis was adjusted for sex, age, family income level, educational level, smoking status, alcohol intake, age at drinking onset, and attempts to reduce or quit drinking (Table 2).

**Table 2:** Association of the alcohol flushing response with depression.

	No depression/depression	Weighted prevalence (%)	AOR (95% CI)^†^	*P*-value
**Alcohol flushing status**				
Never flusher	82,307/1996	2.52	1 (Ref.)	
Former flusher	5951/161	2.66	1.03 (0.83–1.26)	0.812
Current flusher	47,659/1306	2.93	1.23 (1.12–1.34)	< 0.001
**Sex**				
Male	72,559/1288	1.87	1 (Ref.)	
Female	63,358/2175	3.67	3.31 (2.90–3.78)	< 0.001
**Age (years)**				
19–39	38,269/1274	3.41	1 (Ref.)	
40–59	56,374/1103	1.96	0.57 (0.52–0.63)	< 0.001
60–69	23,883/500	2.28	0.48 (0.41–0.57)	< 0.001
≥ 70	17,391/586	3.47	0.60 (0.49–0.73)	< 0.001
**Family income level**				
1st quartile	29,762/1294	5.01	1 (Ref.)	
2nd quartile	20,187/515	3.28	0.65 (0.57–0.75)	< 0.001
3rd quartile	38,736/812	2.36	0.48 (0.42–0.54)	< 0.001
4th quartile	47,232/842	1.91	0.41 (0.36–0.47)	< 0.001
**Educational level**				
None	5950/348	5.97	1 (Ref.)	
Primary	14,113/445	3.83	0.76 (0.62–0.94)	0.010
Secondary	64,568/1627	2.94	0.59 (0.48–0.73)	< 0.001
Tertiary	51,286/1043	2.14	0.48 (0.38–0.59)	< 0.001
**Smoking status**				
Never smoker	78,614/2043	2.71	1 (Ref.)	
Ex-smoker	28,774/513	1.82	1.23 (1.05–1.44)	0.013
Current smoker	28,529/907	3.30	1.95 (1.70–2.23)	< 0.001
**Alcohol intake (g/day)**				
< 5	82,762/2128	2.63	1 (Ref.)	
5–14.9	26,230/628	2.50	0.99 (0.88–1.12)	0.929
15–29.9	17,141/379	2.49	1.02 (0.88–1.18)	0.815
≥ 30	9784/328	3.89	1.60 (1.35–1.89)	< 0.001
**Drinking onset**				
< 19 years old	35,307/1192	3.60	1 (Ref.)	
≥ 19 years old	100,610/2271	2.30	0.61 (0.55–0.67)	< 0.001
**Attempts to reduce/quit drinking**				
No	116,986/2557	2.28	1 (Ref.)	
Yes	18,931/906	4.99	2.14 (1.94–2.37)	< 0.001

AOR, adjusted odds ratio; CI, confidence interval; Ref, reference.

^†^OR adjusted for sex, age, family income level, educational level, smoking status, alcohol intake, age of drinking onset, and attempts to reduce/quit drinking.

### Relationship between the alcohol flushing response and depression according to alcohol intake

Among participants who drank < 5 g and 5–14.9 g of alcohol per day, the risk of depression was significantly greater in current flushers than in never flushers (< 5 g alcohol/day: AOR 1.20, 95% CI 1.07–1.35, *P* = 0.002; 5–14.9 g alcohol/day: AOR 1.39, 95% CI 1.13–1.70, *P* = 0.002). No significant association was detected among individuals who drank ≥ 15 g alcohol/day (15–29.9 g alcohol/day: AOR 1.26, 95% CI 0.95–1.67, *P* = 0.109; ≥ 30 g alcohol/day: AOR 0.97, 95% CI 0.76–1.24, *P* = 0.814 (Table 3).

**Table 3:** Relationship between the alcohol flushing response and depression according to alcohol intake (< 5 g/day, 5–14.9 g/day, 15–29.9 g/day, ≥ 30 g/day) (n = 139,380).

Alcohol intake (g/day)	Alcohol flushing status	AOR (95% CI)^†^	*P*-value
< 5	Never flusher	1 (Ref.)	
	Former flusher	1.07 (0.81–1.41)	0.644
	Current flusher	1.20 (1.07–1.35)	0.002
5–14.9	Never flusher	1 (Ref.)	
	Former flusher	1.06 (0.64–1.75)	0.821
	Current flusher	1.39 (1.13–1.70)	0.002
15–29.9	Never flusher	1 (Ref.)	
	Former flusher	1.00 (0.59–1.71)	0.987
	Current flusher	1.26 (0.95–1.67)	0.109
≥ 30	Never flusher	1 (Ref.)	
	Former flusher	0.85 (0.49–1.47)	0.559
	Current flusher	0.97 (0.76–1.24)	0.814

AOR, adjusted odds ratio; CI, confidence interval; Ref, reference.

^†^OR adjusted for sex, age, social activity, family income level, educational level, smoking status, age of drinking onset, and attempts to reduce/quit drinking.

## Discussion

We investigated the prevalence of the alcohol flushing response and its association with depression in a large community sample of Korean adult drinkers. Of the participants, more than one-third were current flushers; there was a significant link between a current (but not former) flushing response and depression. Current flushers were more likely to develop depression after consuming a small amount of alcohol (< 15 g alcohol/day). The prevalence of the alcohol flushing response was slightly lower (34.8%) than in previous reports (36–45%)^1,2^. The relationship between the flushing response and depression is partially consistent with a report that the alcohol flushing genotype (ALDH2*1/*2) is associated with an increased risk of depression^12^.

Current flushers generally drank less frequently and drank smaller amounts of alcohol, compared with never flushers; most drank < 15 g alcohol/day. This finding is consistent with previous reports in which individuals with inactive ALDH2 had a greater tendency to abstain from drinking^4^. Nevertheless, although the flushers drank a smaller amount of alcohol, they had a comparatively greater risk of depression than did never flushers (AOR 1.23, 95% CI 1.12–1.34, *P* < 0.001). This result contradicts reports that individuals with inactive ALDH2 are protected from alcohol-associated risks because they refuse to drink heavily due to the adverse effects of limited alcohol intake^4^.

The above findings suggest that although flushers tend to drink less, they could be more vulnerable to alcohol-related depression because of the increase of acetaldehyde derived from alcohol in the brain^15^. Excess accumulation of these acetaldehyde may be associated with depression via direct and indirect pathways. Acetaldehyde may directly contribute to anxious or depressive states through the effects of two major stress-related peptides: corticotropin-releasing hormone and neuropeptide Y^10^. These peptides induce aversive states, including a depressive state. Acetaldehyde may indirectly affect depressive status through AUDs. If positive central effects are more pronounced, individuals with inactive ALDH2 may lack protection from alcoholism and may be susceptible to excessive alcohol consumption^15^. The interaction of acetaldehyde with the dopamine system leads to addictive behavior, which is associated with AUDs^10^. AUDs may accompany mental disorders such as depression^10^; they double the risk of major depression^11^. These direct and indirect mechanisms are not restricted to flushers, as they can also be apparent in non-flushers. However, because flushers demonstrate excessive acetaldehyde accumulation despite low alcohol intake^1,5^, they may be affected by such mechanisms after minimal alcohol consumption. It remains to be established whether the biological mechanisms of these direct and indirect pathways exert significant effects in flushers who consume varying amounts of alcohol.

The association of the flushing response with depression was particularly evident in current flushers who drank < 15 g alcohol/day. This finding suggests that, compared with never flushers, current flushers have a lower threshold for the depression risk associated with acetaldehyde exposure. Yokoyama et al.^16^ showed that the blood acetaldehyde level was greater in ALDH2*1/*2 than in ALDH2*2/*2 after low-to-moderate alcohol consumption (median 11 g alcohol/day). Therefore, current flushers have a greater likelihood of experiencing the depressogenic effect of acetaldehyde^10^ at a low level of alcohol intake.

No significant association was found in participants who drank ≥ 15 g of alcohol per day. The lack of a difference between flushers and non-flushers in the risk of depression indicates that internal exposure to acetaldehyde becomes increasingly similar with increasing alcohol intake. Yokoyama et al.^17^ performed a study of alcoholics with high alcohol intake (≥ 80 g in the previous 24 h); they compared blood acetaldehyde levels according to ALDH2 genotype. The blood acetaldehyde level did not significantly differ between participants with ALDH2*1/*2 genotype and participants with ALDH2*1/*1 genotype. Our findings showed that the difference in depression risk between current and never flushers declined at an alcohol intake of ≥ 15 g alcohol/day. Further studies are needed to clarify whether the difference in blood acetaldehyde level between flushers and non-flushers also decreases at an alcohol intake of ≥ 15 g alcohol/day. However, these findings should not be used to encourage flushers to drink ≥ 15 g alcohol/day or to suggest a benefit of drinking alcohol.

Two previous studies used the alcohol flushing response or related genotype as an instrumental variable for lower alcohol intake. Zhu et al.^18^ conducted a Mendelian randomization (MR) study of 476 middle-aged and older Chinese adults (mean age, 49.4 years). They found a protective effect of alcohol on depression when the flushing response was used as an instrument variable. Another MR study of older men in Australia used an ADH1B genetic polymorphism as an instrument variable^19^. In that study, alcohol consumption had no significant effect on depression. MR studies are typically regarded as superior to observational studies; the genetic variants used as instrument variables are inherited and may not be affected by confounders. However, estimates from MR studies can be biased in certain situations^20^. For example, MR studies rely on the assumption that the genetic variant does not affect the disease outcome except through exposure. However, the inactive ALDH2 genotype may increase the risk of depression by pathways other than alcohol exposure, such as pathways that involve endogenous aldehydes^21^. Moreover, inactive ALDH2 may act as an effect modifier that enhances alcohol toxicity in the brain. We did not make the assumptions required for MR studies; however, analyzed the associations between flushing response and depression while controlling for alcohol consumption, along with several potential confounders. MR studies control for unmeasured confounding; our consideration of covariates served the same purpose.

Because cross-sectional secondary analysis was done in this study, there are several limitations. We cannot infer causality between the flushing response and depression, because a temporal sequence could not be established. Additionally, we could not explore some potentially important variables that may have influenced the association between the flushing response and depression because they were absent from the source data. For example, we could not determine the duration of intoxication for each drinking occasion, which is related to the blood alcohol and acetaldehyde levels. We could have evaluated the effects of alcohol-use disorders on the relationship between the flushing response and depression, if we had had access to the appropriate data. Also, bias may have been introduced by unmeasured confounding variables. Further studies of such unmeasured variables could clarify the association. In addition, because this study relied on self-report data, recall bias might have been introduced. Last, we did not compare non-drinkers and drinkers because the question regarding current flushing status was only asked of current drinkers in the 2019 KCHS; such a comparison would have provided insight into any relationship between flushers and depression.

Our results should be carefully compared with other studies on the association between inactive ALDH2 and depression. Although the alcohol flushing response is a marker of inactive ALDH2, for which it has 95.1% sensitivity and 76.5% specificity^22^, the characteristics of individuals with the response may differ from those of individuals with inactive ALDH2. The flushing response may be influenced by environmental factors or genetic traits other than inactive ALDH2^23^. Therefore, further studies are needed to confirm our findings.

Regardless of these limitations, the use of a large, nationally representative sample to address the association between the alcohol flushing response and depression was a major strength of this study. We revealed the distribution of the alcohol flushing response among drinkers in the Korean population, and provide evidence of a relationship between the flushing response and depression. Relative to previous studies that assessed the relationship between the flushing response and depression in a relatively small number of participants, our findings provide more insight into this association.

## Methods

### Study population

This study used data from the 2019 KCHS, which was a community-based, cross-sectional survey conducted by the Korea Disease Control and Prevention Agency. The survey data are suitable for planning, implementing, monitoring, and assessing community health promotion and disease-prevention programs. The survey focused on personal health practices and disease-associated behaviors, such as smoking and alcohol use.

The survey participants were selected from adults (≥ 19 years old, legal age for adults in South Korea) who resided within the catchment area of a community health center. On average, five households were selected at each sampling point; all ≥ 19-year-old members of those households were asked to participate in the survey. Informed consent was obtained from all participants. Two-stage clustered sampling was applied in this study. The first stratum comprised small administrative units, where community health centers are located in South Korea; the second stratum comprised housing units. The sample size of the survey population was determined so that the main health index had a ± 3% sampling error with a 95% confidence level. Data were weighted according to the sample design structure^24^.

Of the 229,099 participants who completed the 2019 KCHS, 82,767 were non-drinkers, while 6952 had missing values regarding the grade of depression, alcohol flushing status, and other covariates. The final study population comprised 139,380 current drinkers. Non-drinkers were either lifetime abstainers or had abstained from alcohol in the past 12 months.

The 2019 KCHS data are publicly available on the KCHS website (http://chs.kdca.go.kr/). Thus, this study was exempt from review by the Institutional Review Board of Seoul National University (IRB E2106/002-002) and was performed following relevant guidelines and regulations.

### Measures

The presence of depression was identified using the PHQ-9, a simple measure that aligns with the DSM-IV criteria^25^. The frequency of depression-related symptoms is rated based on the respondent’s experience over the past 2 weeks, using a four-point scale that ranges from “not at all” to “nearly every day”). The scores are summed as the index of depressive symptoms, with a maximum possible score of 27. A score ≥ 10 was considered indicative of depression.

The alcohol flushing response status was identified based on the responses to the questions: (a) Do you currently tend to flush in the face immediately after drinking as little as a glass of beer (no, occasionally, often, or always)? (b) Did you have a tendency to flush in the face immediately after drinking as little as a glass of beer during the first to second year you started drinking (yes or no)? Respondents who answered “occasionally,” “often,” or “always” to question (a) were classified as “current flushers”; respondents who answered “no” to question (a), but “yes” to question (b) were classified as “former flushers”; and respondents who answered “no” to both questions were classified as “never flushers.” These questions have 95.1% sensitivity and 76.5% specificity for detecting inactive ALDH2 in Koreans^22^.

Alcohol intake as grams of alcohol consumed per day (g alcohol/day) was evaluated by a quantity frequency measure^26^. We calculated the product of the usual number of drinks consumed per occasion (1–2, 3–4, 5–6, 7–9, and ≥ 10 drinks) and the frequency of drinking occasions (less than one time per month, approximately once per month, two to four times per month, two to three times per week, and more than four times per week), then converted the scale from drinks to grams of alcohol (7 g per standard drink in Korea^27^). The quantity and frequency of drinking were assessed for participants who had drunk alcohol in the past 12 months. The values used for quantities and frequencies were the arithmetic mid-points of the number of drinks consumed per occasion (approximate mid-points).

Basic characteristics and variables that show association with depression were set as covariates for the analysis. Respondents’ sex, age, family income level, educational level, obesity, smoking status, exercise, alcohol intake, age at drinking onset, and prior attempt to reduce or quit drinking was considered as either possible intermediates or potential confounders in this study.

Sex was categorized as male or female. Age was categorized as 19–39 years, 40–59 years, 60–69 years, or ≥ 70 years, for even distribution between the age groups. Family income level was categorized into four quantiles. Educational level was categorized as none, primary education, secondary education, or tertiary education. Obesity was categorized as yes or no (body mass index ≥ 25.0 kg/m^2^ or < 25.0 kg/m^2^) according to Asian-Pacific cutoff points^28^. Smoking status was categorized as never smoker, ex-smoker, or current smoker. Exercise was categorized as yes or no according to each participant’s prior exercising habits (vigorous physical activity for more than 20 min for more than 3 days in the past week, or moderate intensity physical activity for more than 30 min for more than 5 days in the past week). Alcohol intake was classified as < 5 g alcohol/day, 5–14.9 g alcohol/day, 15–29.9 g alcohol/day, or ≥ 30 g alcohol/day. The age at drinking onset was divided into < 19 and ≥ 19 years. This was the age that participants recall “drinking more than one standard drink for the first time in their lives.” Prior attempt to reduce or quit drinking was categorized as yes or no, depending on whether the participant had previous attempts to reduce or quit drinking within the past 12 months.

### Statistical analysis

Non-weighted frequencies and weighted percentages were calculated as descriptive characteristics. The chi-square test was used to evaluate differences in demographic, socioeconomic, and health-related variables among the three different flushing status groups (“never”, “former”, and “current”). Multiple logistic regression analysis was used to evaluate the association between alcohol flushing and depression, with adjustment for covariates (sex, age, family income level, educational level, smoking status, alcohol intake, age at drinking onset, and attempts to reduce/quit drinking); The covariates (obesity and exercise) that did not show significant differences (*P* < 0.05) among the flushing groups were not adjusted. In this analysis, the alcohol flushing response was used as the independent variable, and the presence of depressive symptoms (depression) was used as the dependent variable. Their relationship was investigated according to alcohol intake (< 5 g alcohol/day, 5–14.9 g alcohol/day, 15–29.9 g alcohol/day, and ≥ 30 g alcohol/day), with adjustments for confounding variables. Statistical analysis was conducted using SAS ver. 9.4 (SAS Institute, Cary, NC, USA). Two-sided P-values were used, and the level of statistical significance was set at *P* < 0.05.

## Data Availability

The datasets generated and/or analysed during the current study are publicly available in the KCHS repository, http://chs.kdca.go.kr/.

## References

[1] Brooks PJ, Enoch MA, Goldman D, Li TK, Yokoyama A (2009). The alcohol flushing response: An unrecognized risk factor for esophageal cancer from alcohol consumption. PLoS Med..

[2] Enoch MA (2014). Genetic influences on response to alcohol and response to pharmacotherapies for alcoholism. Pharmacol. Biochem. Behav..

[3] Crabb DW, Edenberg HJ, Bosron WF, Li TK (1989). Genotypes for aldehyde dehydrogenase deficiency and alcohol sensitivity. The inactive ALDH2(2) allele is dominant. J. Clin. Invest..

[4] Crabb DW, Matsumoto M, Chang D, You M (2004). Overview of the role of alcohol dehydrogenase and aldehyde dehydrogenase and their variants in the genesis of alcohol-related pathology. Proc. Nutr. Soc..

[5] Mizoi Y (1979). Relationship between facial flushing and blood acetaldehyde levels after alcohol intake. Pharmacol. Biochem. Behav..

[6] Jiang Y, He J, Liu H, Xu Z (2020). Association between ALDH2 rs671 polymorphism and risk of ischemic stroke: A protocol for systematic review and meta analysis. Medicine (Baltimore).

[7] Andrici J, Hu SXH, Eslick GD (2016). Facial flushing response to alcohol and the risk of esophageal squamous cell carcinoma: A comprehensive systematic review and meta-analysis. Cancer Epidemiol..

[8] Shin M-J, Cho Y, Davey Smith G (2017). Alcohol consumption, aldehyde dehydrogenase 2 gene polymorphisms, and cardiovascular health in Korea. ymj.

[9] Yu R-L, Tan C-H, Lu Y-C, Wu R-M (2016). Aldehyde dehydrogenase 2 is associated with cognitive functions in patients with Parkinson’s disease. Sci. Rep..

[10] Brancato A, Lavanco G, Cavallaro A, Plescia F, Cannizzaro C (2017). Acetaldehyde, motivation and stress: Behavioral evidence of an addictive menage a trois. Front. Behav. Neurosci..

[11] Boden JM, Fergusson DM (2011). Alcohol and depression. Addiction.

[12] Yoshimasu K (2016). Depression, alcoholism, and genetic alcohol sensitivity regulated by ALDH2 and ADH1B polymorphisms among japanese community-dwelling adults. Arch. Depress. Anxiety.

[13] Yoshimasu K (2015). Genetic alcohol sensitivity regulated by ALDH2 and ADH1B polymorphisms is strongly associated with depression and anxiety in Japanese employees. Drug Alcohol Depend..

[14] Edenberg HJ, Foroud T (2013). Genetics and alcoholism. Nat. Rev. Gastroenterol. Hepatol..

[15] Correa M (2012). Piecing together the puzzle of acetaldehyde as a neuroactive agent. Neurosci. Biobehav. Rev..

[16] Yokoyama A (2008). Salivary acetaldehyde concentration according to alcoholic beverage consumed and aldehyde dehydrogenase-2 genotype. Alcohol Clin. Exp. Res..

[17] Yokoyama A (2010). Polymorphisms of alcohol dehydrogenase-1B and aldehyde dehydrogenase-2 and the blood and salivary ethanol and acetaldehyde concentrations of Japanese alcoholic men. Alcohol Clin. Exp. Res..

[18] Zhu C (2020). Alcohol use and depression: A mendelian randomization study from China. Front. Genet..

[19] Almeida OP, Hankey GJ, Yeap BB, Golledge J, Flicker L (2014). The triangular association of ADH1B genetic polymorphism, alcohol consumption and the risk of depression in older men. Mol. Psychiatry.

[20] Savla J, Neeland IJ (2018). The Pros and Cons of mendelian randomization studies to evaluate emerging cardiovascular risk factors. Curr. Cardiovasc. Risk Rep..

[21] Cao Y (2021). Neurotoxicity and underlying mechanisms of endogenous neurotoxins. Int. J. Mol. Sci..

[22] Shin CM, Kim N, Cho SI, Sung J, Lee HJ (2018). Validation of alcohol flushing questionnaires in determining inactive aldehyde dehydrogenase-2 and its clinical implication in alcohol-related diseases. Alcohol Clin. Exp. Res..

[23] Yokoyama T (2003). Alcohol flushing, alcohol and aldehyde dehydrogenase genotypes, and risk for esophageal squamous cell carcinoma in Japanese men. Cancer Epidemiol. Biomark. Prev..

[24] Kang YW (2015). Korea community health survey data profiles. Osong Public Health Res. Perspect..

[25] Kroenke K, Spitzer RL, Williams JBW (2001). The PHQ-9. J. Gen. Intern. Med..

[26] Greenfield TK, Kerr WC (2008). Alcohol measurement methodology in epidemiology: Recent advances and opportunities. Addiction.

[27] Kim (2019). Standard drink, guidelines for low risk drinking, and divided discourses. J. Korean Alcohol Sci..

[28] Pan W-H, Yeh W-T (2008). How to define obesity? Evidence-based multiple action points for public awareness, screening, and treatment: an extension of Asian-Pacific recommendations. Asia Pac. J. Clin. Nutr..

